# Supplementary material for: Single cell visualization of transcription kinetics variance of highly mobile identical genes using 3D nanoimaging
Source: Sci Rep. 2015 Mar 19;5:9258. doi: 10.1038/srep09258 (PMC4365385; doi:10.1038/srep09258)
Supplement: Supplementary Information — Supplementary Materials and Discussion [file srep09258-s1.pdf]

Supplementary Materials

Single cell visualization of transcription kinetics variance of highly mobile identical genes using 3D nanoimaging

Paolo Annibale, Enrico Gratton

Supplementary Figures

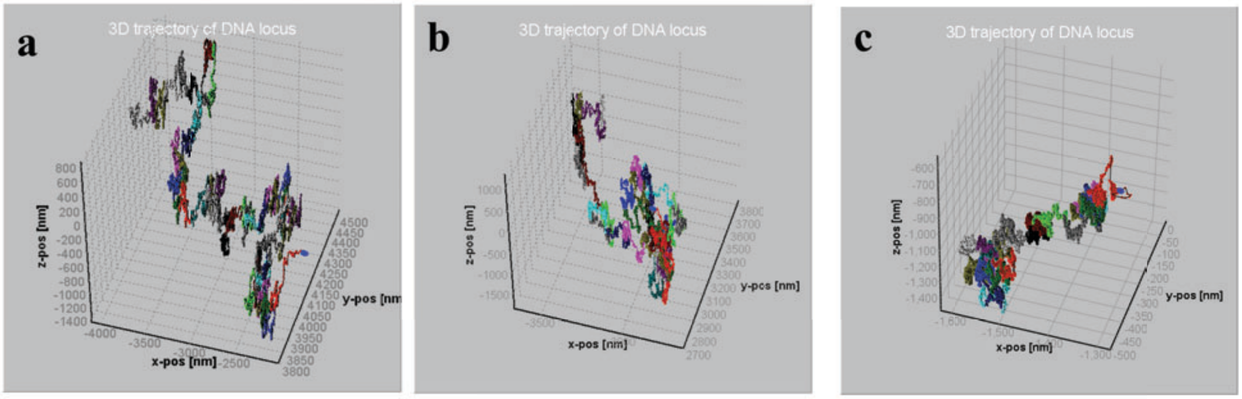

Supplementary Figure 1. Reconstructed 3D trajectory of the fluorescence center of mass of the transgene array within the nucleus of U2OS 263 cells in three representative cases following Doxycycline induction. a) The array displays a maximal displacement of 2 μm along the x direction. b) In this trajectory the maximum displacement occurs along the z direction, for a total of about 2.5 μm. c) Trajectory of a transgene array following 30' minutes incubation with 10 μM Sodium Azide and 50 μM 2-Deoxyglucose. The maximal displacement has been reduced to sub-μm size.

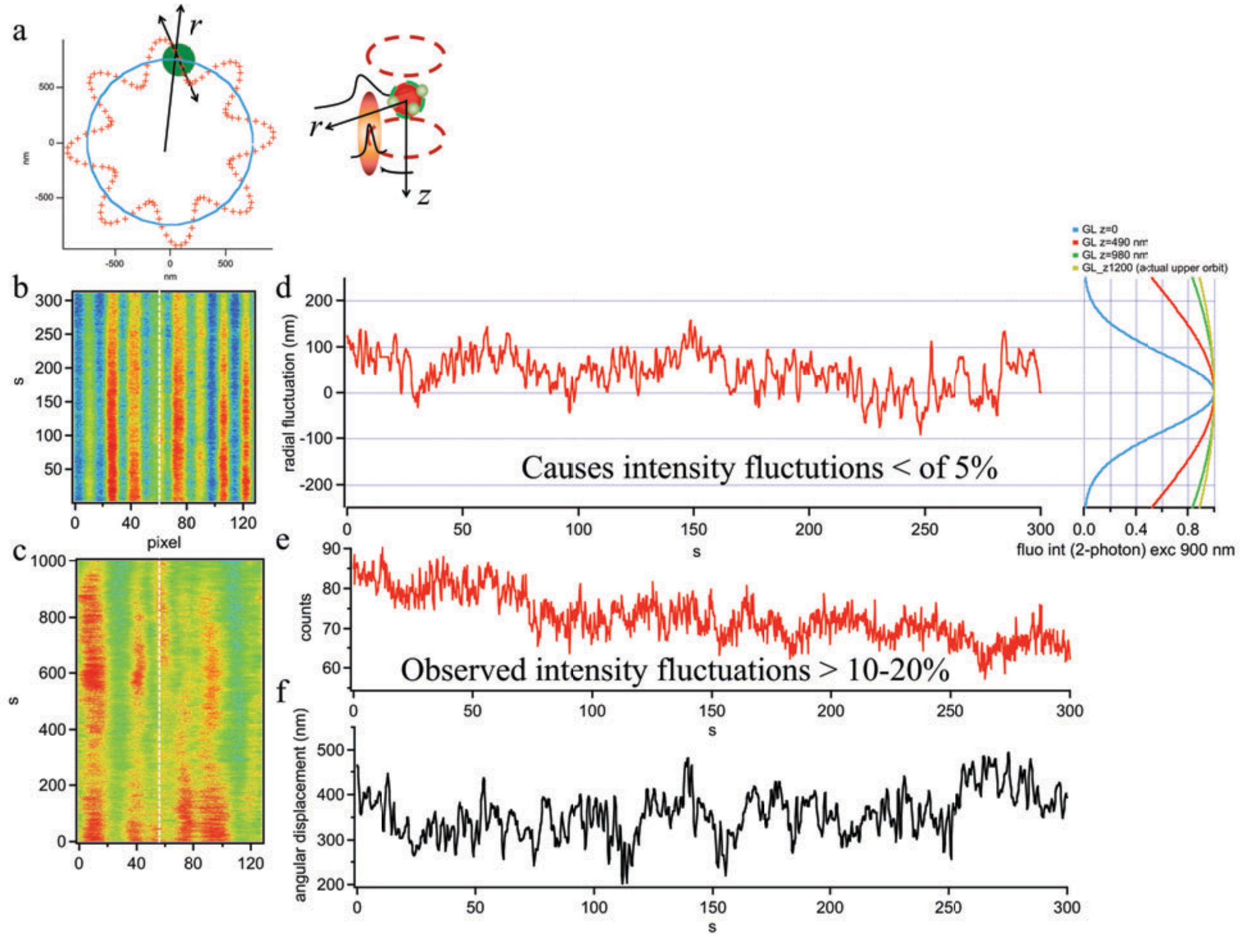

Supplementary Figure 2 Effect of the spatial fluctuations of the petals on the fluorescence intensity traces collected performing orbital tracking. The fluctuations of the petals are predominantly angular. The contribution of radial fluctuations can be examined by switching from a circular orbit to a Rosetta shape. a) A Rosetta orbit intersecting a petal allows monitoring the radial component of the displacement of the petals. In 3D orbital tracking geometry, only the distal portion of the PSF along the z-axis intersects the petals. b) Intensity carpet collected along a Rosetta orbit and c) intensity carpet collected, shortly after, along a circular orbit surrounding the same transgene array. d) The radial displacement of a representative petal (in nm) is displayed together with the profile of the Gaussian-Lorenzian Point Spread Function of the 2-

Photon microscope for an excitation wavelength of 900 nm. According to the relative z-distance of the orbit with respect to the plane of the petals, the PSF broadens along the radial direction. For the typical distance of the upper/lower orbits from the center of the transgene array of approximately 1 μm, the PSF is so broad that the intensity fluctuations caused by radial oscillations would contribute less than 5% of the total intensity. e) Intensity fluctuations collected during a circular scan on the same petal as in d. f) Angular displacement of the petal along the circular orbit.

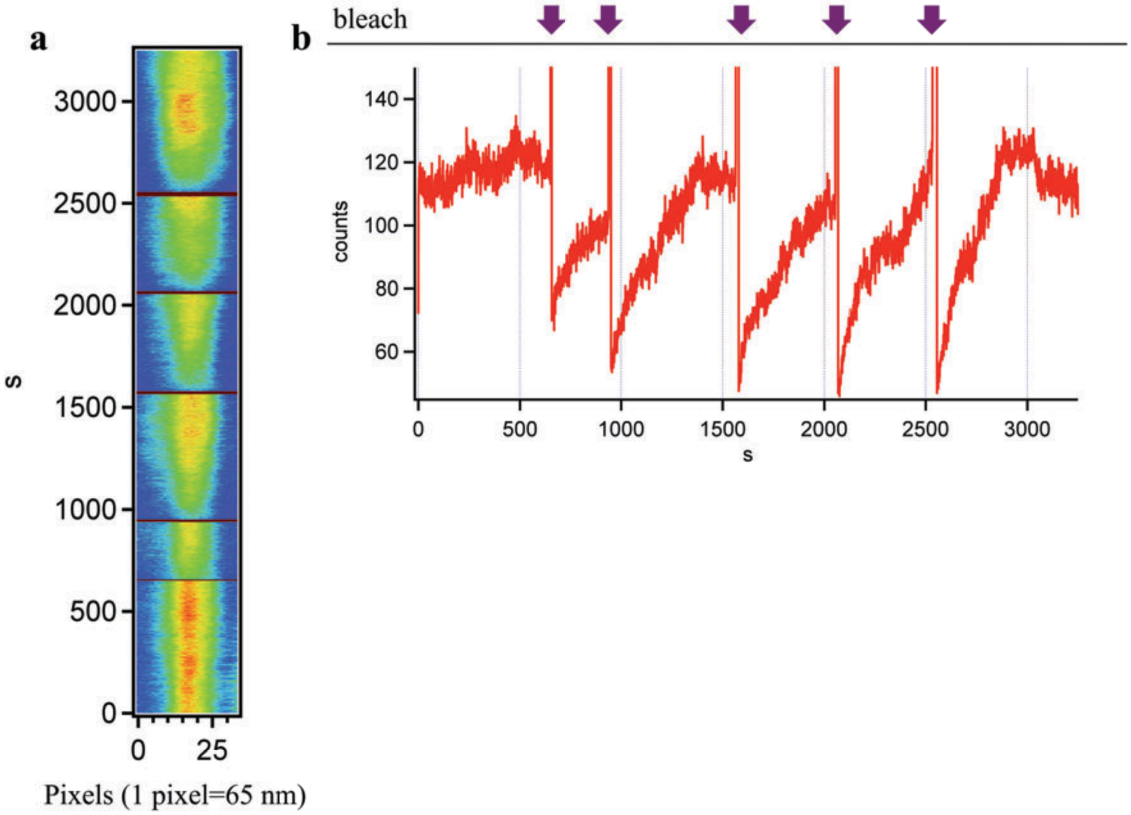

Supplementary Figure 3. Tracking-FRAP of an active petal a) Intensity carpet and average fluorescence trajectory of a *petal* subjected to short pulses of intense excitation light to cause photobleaching of the fluorescently labeled mRNA molecules. The unit of the lower axis is pixels along the orbit. b) Recovery of the fluorescence intensity at each cycle confirms that the petal is a genetic locus undergoing active transcription.

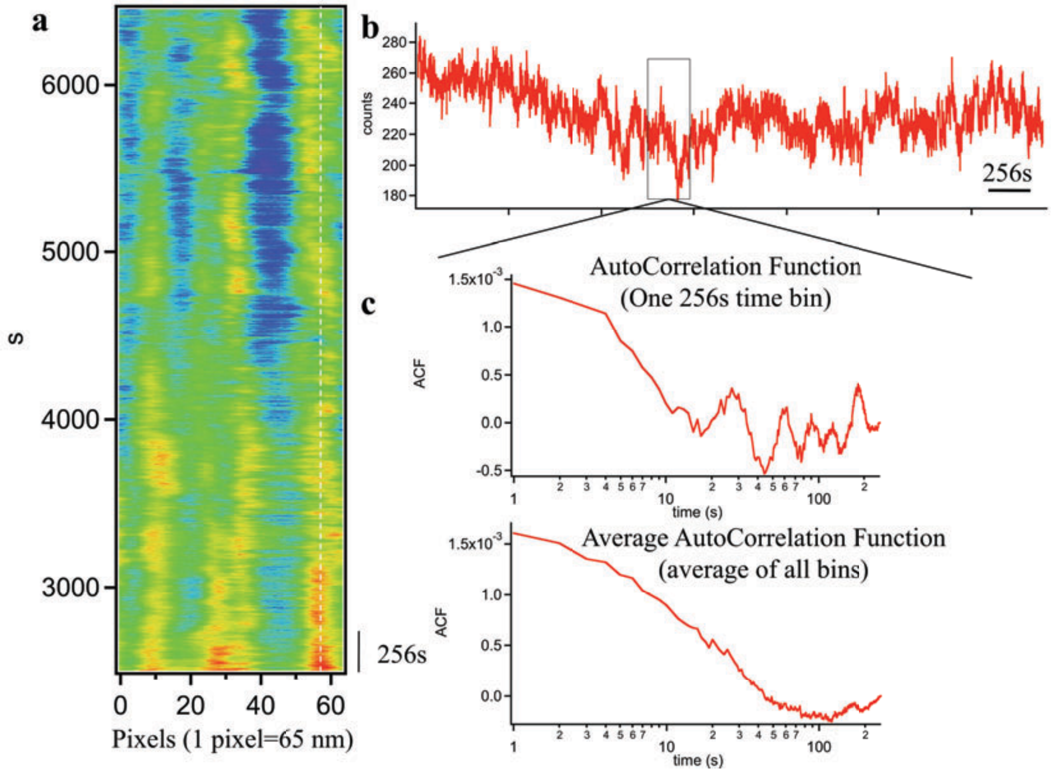

Supplementary Figure 4. Autocorrelation analysis of the petal carpets a) Intensity carpet and b) Fluorescence trajectory. c) AutoCorrelation (ACF) function calculated starting from short time bins. In this example time bins of 1024 points (256 s) are used. The global autocorrelation function of an active petal is calculated by averaging all the ACFs of individual bins.

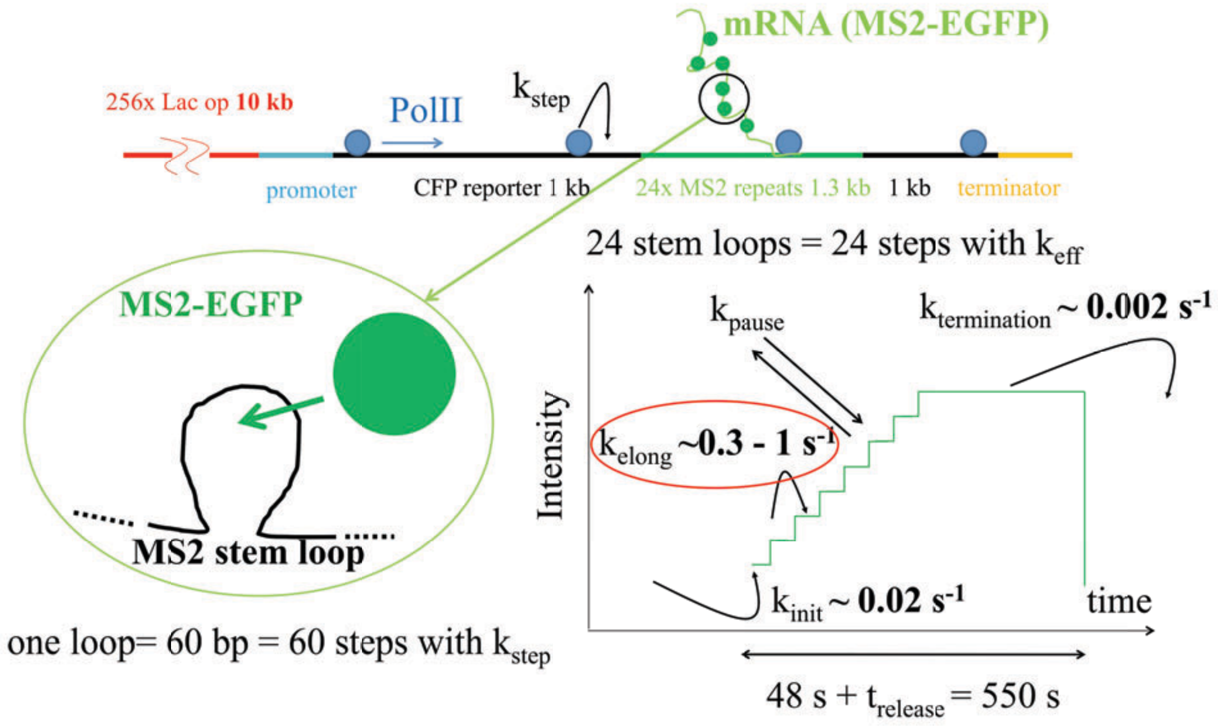

Supplementary Figure 5. Kinetic model for the analysis of MS2-mRNA fluorescence traces.

a) Diagram of an individual repeat within the U2OS transgene array, with annotated length of each relevant portion (reporter gene, MS2 cassette, downstream sequence). b) The periodicity of the MS2 stem loops is about 60 bp, and the MS2-GFP construct binds as a dimer. c) Changes of fluorescence intensity on the gene due to the synthesis of the mRNA molecule. Kinetic rates are taken from the work of Darzacq *et al* [1].

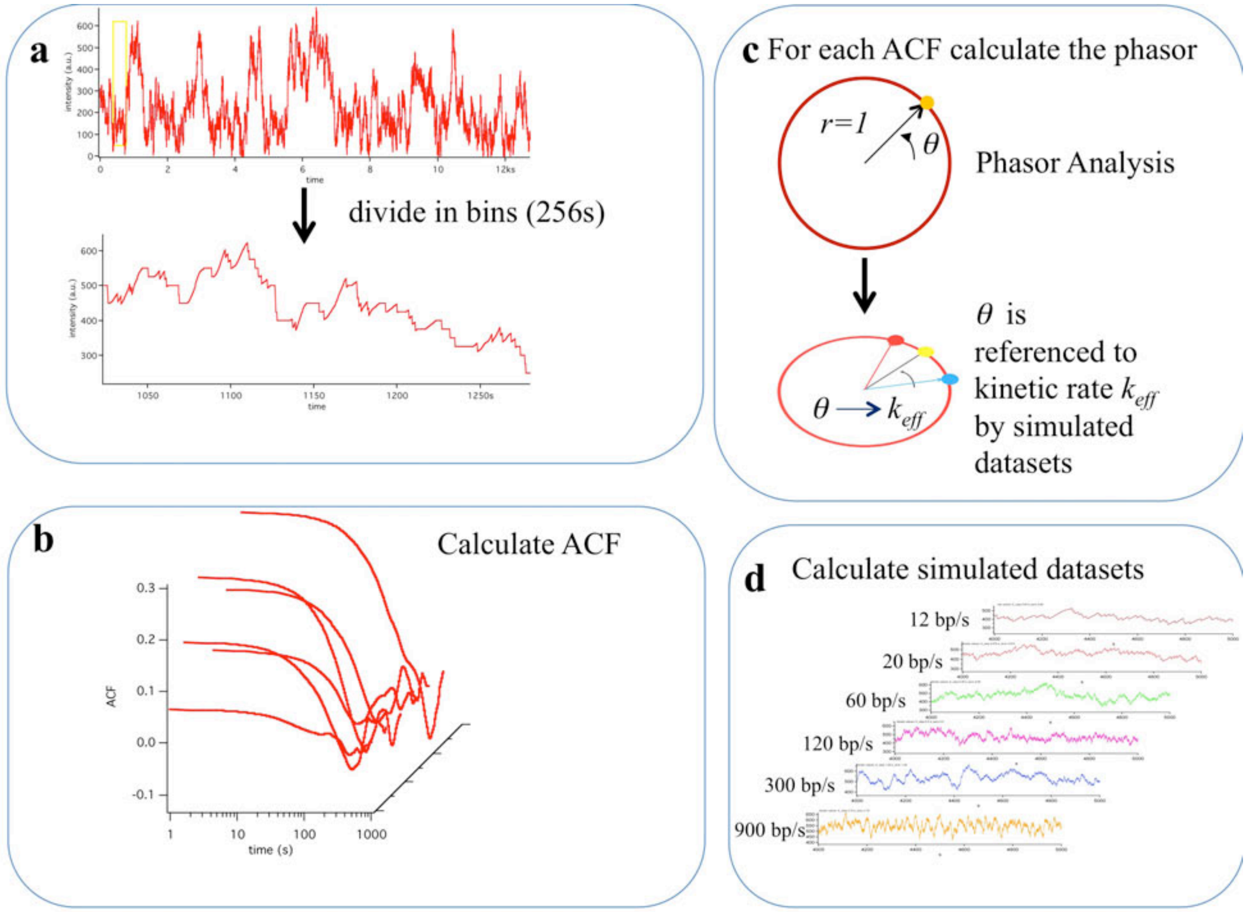

Supplementary Figure 6. Phasor analysis of the experimental data. a) The fluorescence trajectory (here a simulated dataset) of an individual petal is divided in time bins (N=1024 pts = 256 s) and the b) Autocorrelation Function (ACF) of each is calculated. c) The phasor analysis of the autocorrelation function converts the kinetic information within the ACF into an angle in the complex plane. The experimental points can be referenced using simulated datasets. d) Simulated trajectories are calculated for varying kinetic parameters (elongation rate, termination rate).

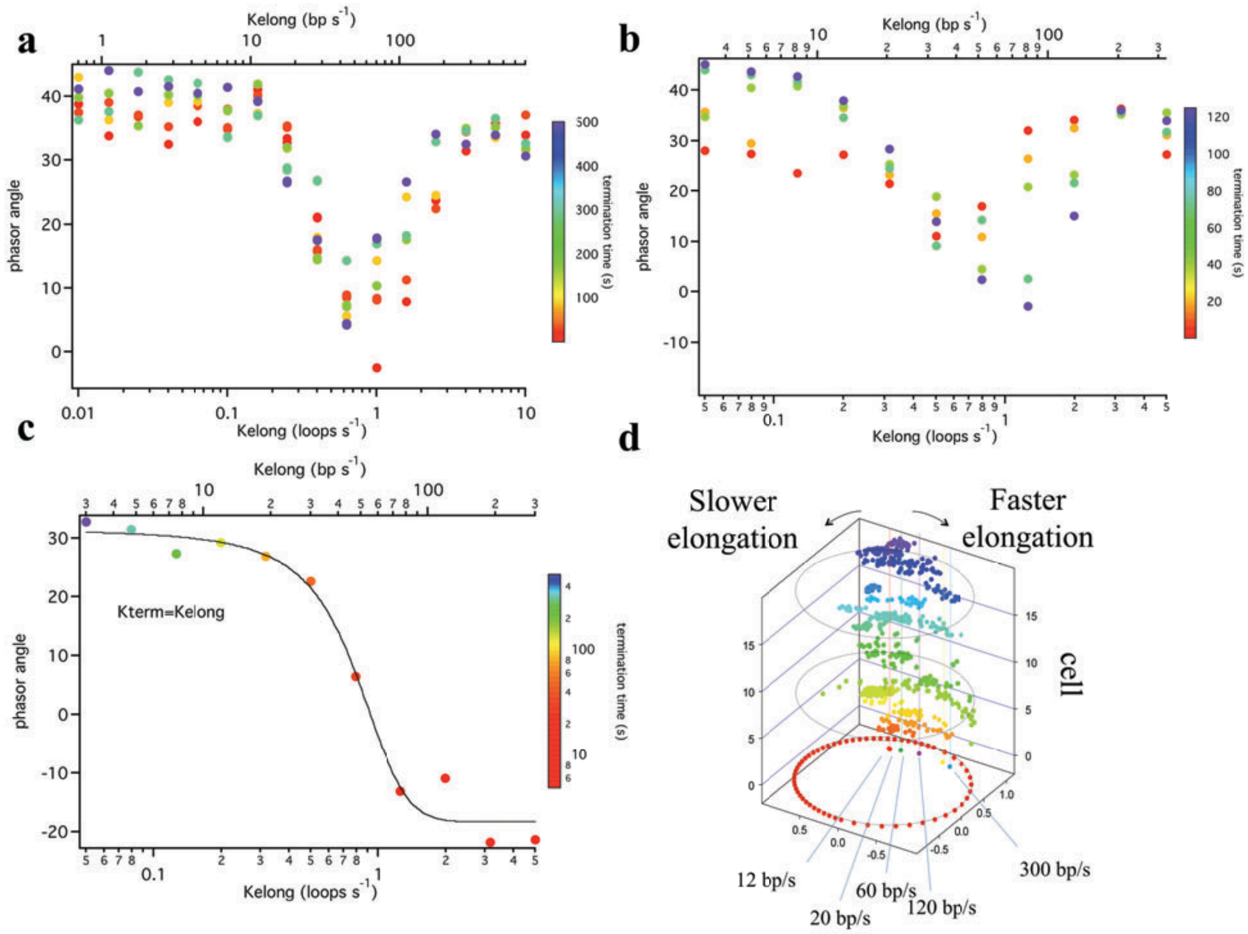

Supplementary Figure 7 Phasor analysis of simulated and experimental datasets. a) Phasor angle (degrees) as a function of the elongation rate (k_{elong}) for varying average termination rates, assuming an exponential distribution of termination rate. b) Phasor angle as a function of the elongation rate (k_{term}) for varying termination rates assuming a fixed termination time. c) Dependence of the phasor angle upon the elongation rate assuming k_{elong}=k_{term} , viz that PolII runs through the last 60 bp of the gene at the same speed it does on the MS2 cassette and is then instantly released. d) 3D scatter plot of experimental phasor points calculated from the fluorescence kymographs of petals in different cells. Reference phasor points are indicated at the bottom of the graph, according to the calibration curve in panel c.

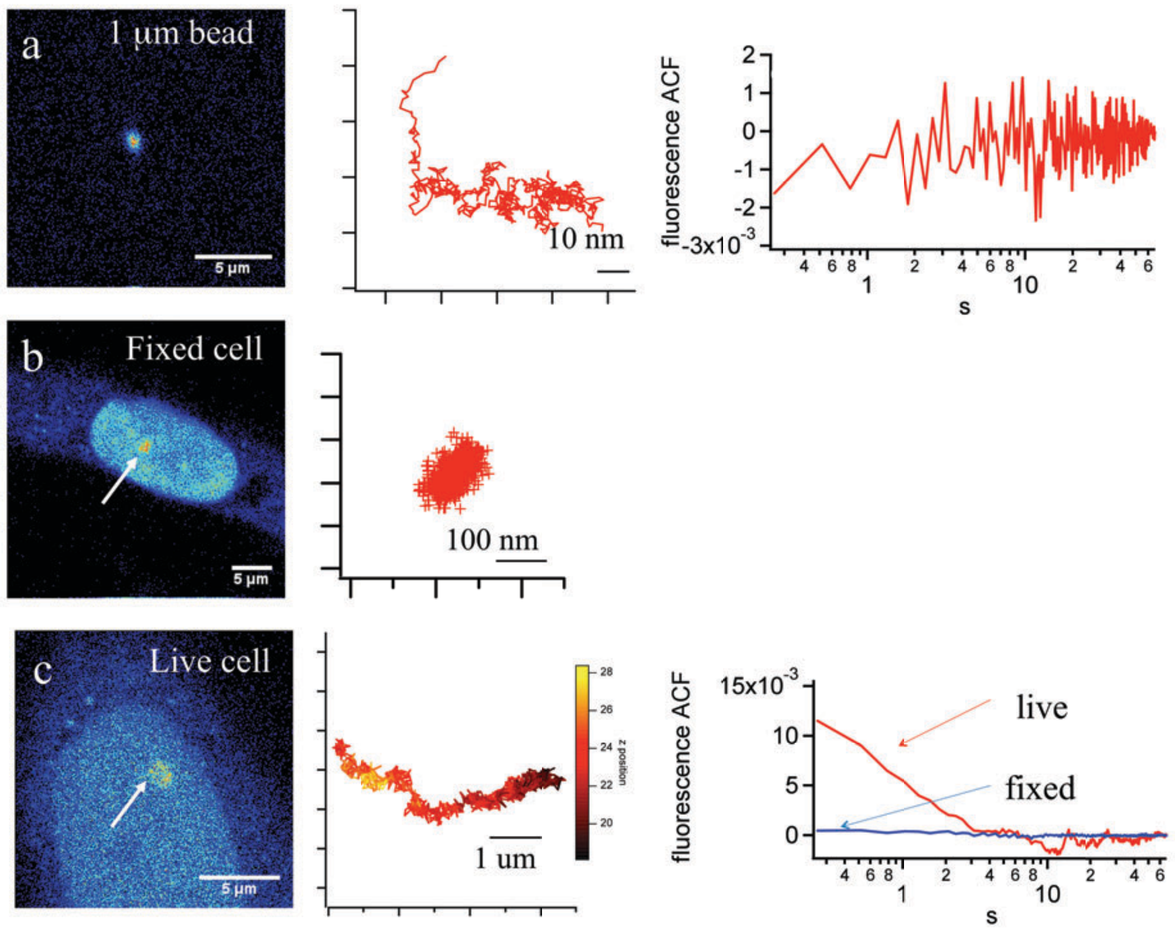

Supplementary Figure 8 Control experiments. a) Tracking of an immobile 1 µm bead on a glass surface. Raster scan image (left), x-y projection of the 3D trajectory (center) and autocorrelation function of the fluorescence intensity (right). b) Tracking of an inducted array within a fixed cell. Raster scan image (left) and x-y projection of the 3D trajectory (right). c) Tracking of an inducted array within a living cell. Raster Scan image (left), x-y projection of the 3D trajectory (center) and Autocorrelation function of the fluorescence intensity collected from the MS2-EGFP constructs compared to the ACF collected on a fixed cell (right).

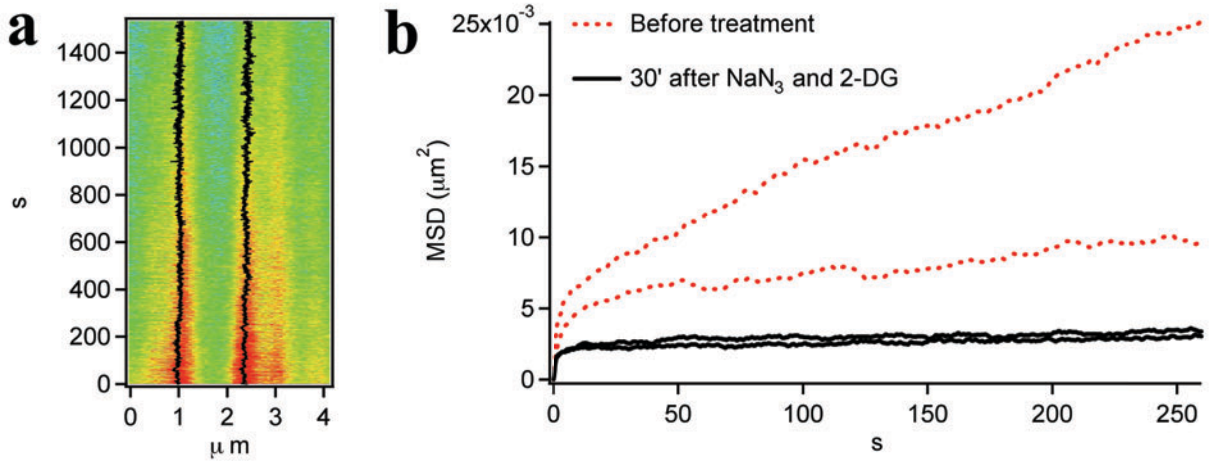

Supplementary Figure 9 ATP depletion treatment a) Representative intensity carpet displaying the fluorescence of mRNA petals surrounding the transgene array in a cell treated with 10 mM Sodium Azide and 50 mM 2-Deoxyglucose for 45’. Angular fluctuations in the petals motion are effectively suppressed. The progressive decrease in fluorescence intensity of each petal is due to photobleaching of the MS2-EGFP, not compensated by the synthesis of new mRNAs. b) Comparison of the Mean Squared Displacement (linear scale) calculated on two petal trajectories before and two trajectories after ATP depletion. ATP depleted trajectories display a minimal residual motion, indicating a corralling size $(MSD)^{0.5}$ of about 95 nm, corresponding to 1.5 times the pixel size of the measurement.

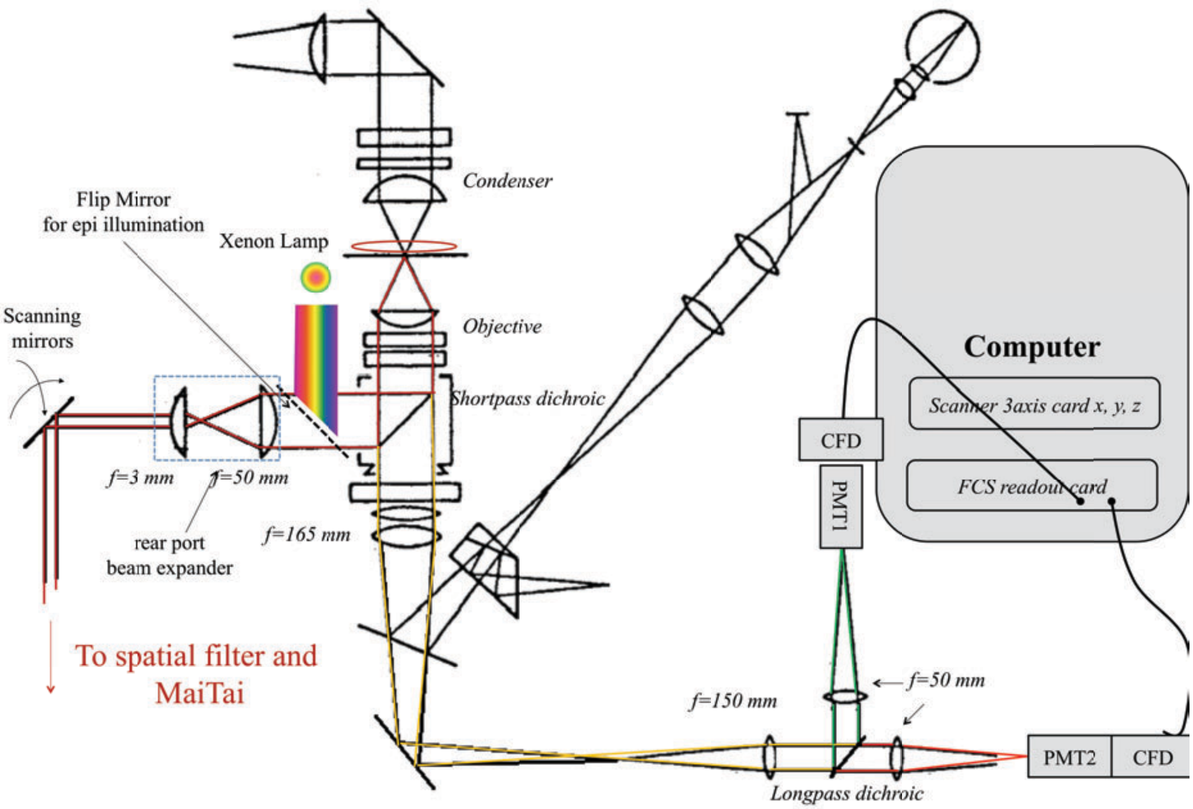

Supplementary Figure 10 Schematics of the tracking microscope. The expanded and collimated infrared beam from an Ti:Sa Laser (MaiTai, Spectra Physics) is coupled into the rear port of a Zeiss Axiovert 135 inverted microscope. A pair of scanning mirrors placed in the focus of a beam expander allows steering the beam in the sample plane. A 60X 1.2 NA Olympus water objective is used. The fluorescence is collected through a dichroic short-pass filter and split between two detectors using an additional long-pass dichroic mirror. The signal from the photomultiplier tubes (PMT) is amplified, discriminated (CFD) and the digital output is read out using a fast FCS data acquisition card.

Supplementary Materials and Discussion

Experimental setup

The excitation laser beam was provided by a MaiTai Ti:Sa (Spectra Physics) laser source, providing pulsed IR excitation tunable in the range between 690 and 1040 nm. The power of the excitation beam is controlled using an AcoustoOpic Modulator (AOM) MT110 (AA Optoelectronics, Orsay, France). The first order beam output from the AOM is beam expanded to approximately 0.5 inches and spatially filtered through a 15 μm pinhole (Thorlabs).

Microscopy experiments were performed on a Zeiss Axiovert 135 microscope frame, modified for orbital particle tracking as illustrated in **Supplementary Figure 10**. The rear port was fitted with a customized aluminum lens holder to accommodate a short focal distance lens (f=3 mm, a microscope eyepiece) and a collimating f=50 mm lens mounted on a 1 inch cage system (Thorlabs). The two lenses act as an effective beam expander able to overfill the back focal plane (BFP) of the microscope objective (Olympus 60X, 1.2 NA Water). The beam is reflected into the objective BFP after being reflected by a 680 nm short pass dichroic mirror (Semrock). A 1-inch flip mirror (Thorlabs) was inserted after the collimating lens to switch from laser excitation to wide-field excitation using the slightly defocused beam originating from a Xenon Lamp.

A pair of Galvanometer Scanning Mirrors (Cambridge Technologies) was installed in the focal point of the f=3 mm lens (a plane in the Fourier Space with respect to the sample plane, hence a change in angle reflects in a change in lateral position in the sample plane) in order to scan the beam in the sample plane. The scanners were mounted and adjusted on the optical axis of the

rear port beam expander using a pair of two inches steel posts that were then clamped to the optical table.

The emitted fluorescence is collected through the bottom port of the Axiovert 135 frame, and re-collimated using a 150 mm achromatic doublet (Thorlabs). The collimated fluorescence is split through a 565 longpass dichroic mirror (Chroma) and bandpass filtered (510-20 nm and 610-50 nm, Chroma) to be directed to two Hamamatsu H7422P-40 Photomultiplier Tubes. Detection is performed in photon counting mode, and the signal output from each Photomultiplier tube is electronically amplified (ISS 40493) and discriminated using a Constant Fraction Discriminator model 6915 (Phillips Scientific). The TTL output of the discriminator is supplied to a fast readout card FCS 1.1 PCI Card (ISS) with a maximal acquisition frequency of 500,000 Hz.

XY translation of the sample is achieved using an MS2000 stepper motor stage (ASI), with a controller for the coarse axial positioning of the objective turret. Furthermore, the objective is installed on an A260 *Z*-axis piezoelectric nano-positioner stage (ISS).

The voltage to control the Galvo Scanners and the nanopositioner Stage is provided using an ISS 3-axis PCI card. The sample is kept at 37 °C by the use of a PDMI-2 MicroIncubator (Harvard Apparatus), adapted onto the MS2000 stage. The entire data acquisition via the FCS card and the control of the scanning microscope via the 3-axis card is performed using the SimFCS® software, developed by Enrico Gratton at the Laboratory for Fluorescence Dynamics.

MonteCarlo Simulations of Pol II motion along the gene and generation of the fluorescence trajectory

We run simulations of fluorescence trajectories of the MS2-EGFP signal upon a given set of kinetic parameters for PolII elongation on our gene (**Supplementary Figure 5**). In our case, the use of simulated datasets is necessary to provide an absolute calibration to the phasor plot values. As discussed in the main text, the phasor plot of the data can tell us that different petals display markedly different elongation kinetics, but cannot provide us with absolute values unless the elongation rate of the PolII were known beforehand. For this reason we had to recur to simulated trajectories for varying values of the elongation rate and the termination rate to calculate the reference phasor points that are displayed in **Supplementary Figure 6d** and used to obtain the kinetic values in reported in **Figure 3**.

The final observable in the simulated experiment is the fluorescence intensity, increasing from the addition of MS2 subunits to the nascent mRNA and decreasing by the detachment of complete mRNA molecules. Each mRNA molecules carries 24x MS2 stem loops, each of them can be bound by an MS2-EGFP dimer. For simplicity we have assumed that all the loops are occupied (although we are well aware that Wu et al. [2] demonstrated that for the MS2 system only half of the sites end up being occupied, even at high MS2-EGFP monomer concentrations) and that the dissociation constant for MS2-EGFP to the mRNA stem loops is negligible. In each gene of the trans-gene array of U2OS 263 the 24x MS2 cassette is about 1.5 kb long and is followed by approximately another 1 kb of downstream gene before the PolyA site.

We model the gene as a sequence of units, each 60 bp long, corresponding to the approximate length of an MS2 stem loop. The first 24 of these units are active, meaning that once occupied by

a PolII one unit of fluorescence is added to the total fluorescence at that time point, while the remaining 16 do not give rise to any fluorescence. Once a PolII reaches the end of a gene the total fluorescence is diminished of 24 units. For modeling purpose we neglect the portion of the gene before the MS2 cassette, since it does not give rise to any fluorescence signal. A first order kinetics is used to model the stepping forward of PolII from one of these units to the next one. This is along the lines of what reported in Larson et al. [3] but differs significantly from other approaches, such as the TransWave model employed by Maiuri et al. [4]. If a PolII occupies a site, upstream PolII enzymes cannot occupy that site, hence, if a PolII pauses, a pile-up of trailing enzymes is formed. An effective kinetic constant, that is an effective elongation rate is used and is a parameter that can be tuned within the model. First order kinetics is also used to model PolII initiation, release from the gene and transition and recovery to a paused state.

In the current report we followed two fundamental assumptions that were previously employed by [3] in deriving an analytical model for autocorrelation of PolII elongating along MDN1 gene in yeast. First, the mRNA release constant was assumed identical to the effective elongation rate. This is a significant assumption, since a slow release of the mRNA from the gene could be the rate-limiting step. However, as illustrated in **Supplementary Figure 7 a-b**, even significant variations of the PolII termination rate do not impair the ability to detect changes in elongation rate in the relevant range 8-80 bp/s. Second, PolII is assumed not to pause while transiting over the MS2 cassette.

Accepting these two assumptions, we are left with only two parameters to set, initiation and effective elongation. We inserted the initiation rate in our model as a constant, using the value reported by Darzacq et al. of 0.0216 /s. Changing the initiation value did not affect significantly

the result of our analysis. The elongation rate was then varied between 10 bp/s and 900 bp/s, giving rise to the simulated trajectories reported in **Supplementary Figure 6d**.

It should be further noted that if the assumption by Larson *et al.* [3] that the termination rate and the effective elongation rate are equal does not hold (i.e. $k_r \neq k_s$), then the elegant form of the autocorrelation function for short genes determined by the authors,

$$G(\tau) = \frac{k}{c}\left(\frac{2}{3}\right)\frac{1}{\left(N(N+1)\right)^2} e^{-k\tau}\sum_{n=0}^{N}(N-n)(N-n+1)(2N+n+1)\frac{(k\tau)^n}{n!}$$

Equation 1

turns into a considerably more complex expression. For the simpler case of only 5 stem loops, we have obtained an analytical equation for the autocorrelation function (posing the elongation constant $k=k_s$, and defining a termination constant k_r):

$$G(t) = \frac{k_i}{6k_r k_s\left(k_r - k_s\right)^4}\left\{e^{-k_s t}\left[t^3\left(k_r k_s^7 + k_r^2 k_s^6 - 9k_r^3 k_s^5 + 11k_r^4 k_s^4 - 4k_r^5 k_s^3\right) + t^2\left(12k_r k_s^6 + 12k_r^2 k_s^5 - 93k_r^3 k_s^4 + 102k_r^4 k_s^3 - 33k_r^5 k_s^2\right) + \ldots\right]\right\}$$
$$\ldots + t\left(60k_r k_s^5 + 60k_r^2 k_s^4 - 390k_r^3 k_s^3 + 390k_r^4 k_s^2 - 120k_r^5 k_s\right) + \left(120k_r k_s^4 + 120k_r^2 k_s^3 - 630k_r^3 k_s^2 + 600k_r^4 k_s - 180k_r^5\right)\ \Big] + \ldots$$
$$\ldots + e^{-k_r t}\left(-150k_s^5 + 300k_r k_s^4 - 300k_r^2 k_s^3 + 150k_r^3 k_s^2 - 30k_r^4 k_s\right)\ \Big\}$$

Equation 2

Which in our hands was not amenable to further simplification.

Analysis of petals intensity carpets:

As discussed in the main text, the fluorescence intensity collected in the EGFP channel of a tracked active locus can be represented in the form of an intensity carpet, where each line represents the fluorescence intensity collected along the orbit (in most experiments 0 to 64 points

corresponding to 0 to 360 degrees). Each line is collected at a time interval dictated by the temporal resolution of the experiment, and as illustrated in **Figure 1c**, the angular motion of each *petal* is reflected in a zigzag appearance of the intensity trace. In order to perform fluctuation analysis of the signal collected along the columns two corrections to the raw data need to be performed. First, the angular displacement of the trajectory over time needs to be corrected. We perform this by extracting sub-carpets that fully contain the intensity trace of only one petal. Each line of this petal carpet is fit by a 1D Gaussian Function to localize its center and the width of the trace. Initial guesses for the fit of line *k* are estimated by calculating the fit over the integral of a set of *m* preceding lines, from *k-m* to *k.* This provides a memory or *inertia* to the fitting algorithm, as implemented in SimFCS (Globals Software, Enrico Gratton and the Laboratory for Fluorescence Dynamics). The typical memory *m* that is used for the fit is 128 lines, corresponding to about 33 s at the typical acquisition frequency of 3.85 Hz.

Second, it is necessary to correct for trends in the fluorescence intensity over long time scales, such as photobleaching or decline in MS2-EGFP signal following transcription inhibition. However, as the signal intensity decays following perfusion of ActinomycinD (to inhibit transcription), it was necessary to correct the data. A moving average correction, calculated on the carpet of each petal and with a size of 1024 points=256 s was employed in this case.

pCF analysis

pCF was performed, as previously reported, using the SimFCS software, on the intensity carpet collected while performing tracking of a chromatin array. The voltage supplied to the galvanometer mirrors was adjusted in order to obtain a three-lobes shape, the trefoil illustrated in **Figure 2**, instead of a circular orbit.

Phasor Analysis:

Phasor analysis has been successfully used in the context of lifetime and spectral imaging, and we provide here for the first time an extension to the analysis of in-vivo enzyme kinetics.

Phasors of the experimental dataset were calculated from the petal intensity carpet according to the following strategy: each petal is divided into bins of 256 s (1024 points) and the fluorescence autocorrelation function (ACF) of each column of the petal-bin is calculated. The fluorescence ACFs calculated in each of the columns is then averaged to yield a global bin-petal ACF.

The phasor analysis is performed on this function as previously described [5]. Briefly, the FFT of the data is calculated and the real and imaginary parts of the first harmonic are used to calculate the modulus and the angle of a point in the complex space. In the current analysis, where we are ultimately interested in the rate constant, i.e. the slope of the autocorrelation function, only an angle is calculated, and therefore all the points lie on a unit-radius circle. Furthermore, this method acts as a high-pass filter on the data, and fluctuations on timescales longer than 256 s (<0.008 Hz) are effectively rejected. The process is schematically illustrated in **Supplementary Figure 6a-d**.

The phasor analysis of the autocorrelation curves as the one displayed in **Supplementary Figure 4c** is performed according to the standard equations introduced in the past for lifetime or spectral analysis [6], [5], [7]. For each autocorrelation curve described by the function *F(t)* a point on the unit circle in the complex plane is calculated using the *S* and *G* coordinates, according to

$$S(\omega_1) = \frac{\int F(t)\sin(\omega_1 t)dt}{\int F(t)dt}$$

$$G(\omega_1) = \frac{\int F(t)\cos(\omega_1 t)dt}{\int F(t)dt}$$

Equation 3

$\omega_1 = \frac{1}{2N\Delta t}$ is the fundamental frequency and is determined by the temporal resolution *Δt* of our measurement and by the time bin used to calculate the ACF, typically 256 s yielding *N=1024* .

The angle ϕ of each *phasor* component is given by: $\phi = a\tan(\frac{S}{G})$. In practice *S* and *G* are calculated using a Fast Fourier Transform (FFT) of the Auto Correlation Function and posing:

$$G(k) = \mathrm{Re}\frac{FFT(k) + FFT(N-k)}{(\sqrt{\mathrm{Re}(FFT(k))^2 + \mathrm{Im}(FFT(N-k))^2}}$$

$$S(k) = \mathrm{Im}\frac{FFT(k) + FFT(N-k)}{(\sqrt{\mathrm{Re}(FFT(k))^2 + \mathrm{Im}(FFT(N-k))^2}}$$

Equation 4

where *k* is the discrete frequency index corresponding to the continuous variable ω_1 used in the previous formulas. *k=1* yields the first harmonic of the measurement, *k=2* the second harmonic and so forth.

In being a fit-less approach, phasor analysis requires a calibration to extract quantitative information from the data. We chose to calibrate the kinetic phasor using MonteCarlo generated PolII fluorescence trajectories.

It is worth noticing that the reference phasors depend upon the model and upon the parameters (such as initiation, elongation and termination constants) chosen to run the simulations. Upon changing the model, the absolute position of the experimental phasor would map to a different set of kinetic parameters; however, the observation of a relative difference between any two experimental datasets is not model dependent.

The obvious advantage of this approach is that it provides an immediate and graphical way to compare elongation rates across multiple datasets, capturing relative differences without the constraint of having to fit a model to the experimental data. We should also note here that elongation rates slower than about 10 bp/s and larger than about 200 bp/s are more difficult to resolve using the current binning interval of the fluorescence trajectories (1024 points=256 s) **Supplementary Figure 7c**, to the point that the 12 and 20 bp/s reference phasors almost overlap, as illustrated in **Supplementary Figure 7d**. Furthermore, the general trend of a clockwise increment of the elongation rates (reduction in phasor angle) holds true up to about 240 bp/s. This should be ascribed to the fact that above 240 bp/s the addition of new MS2 subunits goes beyond the temporal resolution of the measurement. Therefore in the current implementation the method appears to be able to resolve elongation rates over a one order of magnitude range between 20 bp/s and 240 bp/s.

By construction, all of the points fall on a circle (plus or minus a radial jitter to facilitate the display of the data), and the kinetic fingerprint of each of the petals is mapped into an angular value

References

1. Darzacq X*, et al.* In vivo dynamics of RNA polymerase II transcription. *Nature structural & molecular biology* **14**, 796-806 (2007).

2. Wu B, Chao JA, Singer RH. Fluorescence fluctuation spectroscopy enables quantitative imaging of single mRNAs in living cells. *Biophysical journal* **102**, 2936-2944 (2012).

3. Larson DR, Zenklusen D, Wu B, Chao JA, Singer RH. Real-time observation of transcription initiation and elongation on an endogenous yeast gene. *Science (New York, NY* **332**, 475-478 (2011).

4. Maiuri P*, et al.* Fast transcription rates of RNA polymerase II in human cells. *EMBO Rep* **12**, 1280-1285 (2011).

5. Digman MA, Caiolfa VR, Zamai M, Gratton E. The phasor approach to fluorescence lifetime imaging analysis. *Biophysical journal* **94**, L14-16 (2008).

6. Redford GI, Clegg RM. Polar plot representation for frequency-domain analysis of fluorescence lifetimes. *J Fluoresc* **15**, 805-815 (2005).

7. Andrews LM, Jones MR, Digman MA, Gratton E. Spectral phasor analysis of Pyronin Y labeled RNA microenvironments in living cells. *Biomed Opt Express* **4**, 171-177 (2013).
